# A pathogen-detection’s odyssey in a case of skull base osteomyelitis: Land ahoy!

**DOI:** 10.1186/s12941-025-00796-6

**Published:** 2025-04-26

**Authors:** Laurenz Althaus, Insa Joost, Katharina Schaumann, Tom Prinzen, Maika Werminghaus, Susann Thyson, Birgit Henrich, Jörg Schipper, Thomas Klenzner

**Affiliations:** 1 Department of Otorhinolaryngology, Medical Faculty and University Hospital Düsseldorf, Heinrich-Heine University, Düsseldorf, Germany; 2 Institute of Medical Microbiology and Hospital Hygiene, Medical Faculty and University Hospital Düsseldorf, Heinrich-Heine University, Düsseldorf, Germany

## Abstract

**Background:**

Skull base osteomyelitis (SBO) is a severe disease not only because of its rapid progression and its high mortality: diagnosis and treatment are often protracted and in more than 30% of cases no causative pathogen can be detected. SBO is usually preceded by immunodeficiency, which is why opportunistic infections caused by atypical pathogens must also be taken into consideration. In consideration of the different possible entities, an interdisciplinary approach with surgical debridement, pathological sampling, microbiological testing and antimicrobiological therapy is indispensable.

**Case presentation:**

We report on a 58-year-old female patient who presented to our clinic for the first time in 2014 with a bilateral skull base osteomyelitis. The patient had a history of several comorbidities, including hypogammaglobulinemia following the successful treatment of a relapsed B-CLL. Different surgical treatments had already been attempted at the time of initial presentation. Several rheumatological, orthopedic, haemato-oncological and divergent microbiological differential diagnoses could be ruled out. Despite various interdisciplinary treatment attempts (including surgery, antibiotic therapies and hyperbaric oxygen therapy) the progress led to a palsy of the caudal cranial nerve group in 2022. With all preceded microbiological sampling being negative, we initiated species specific PCRs covering atypical organisms. An atypical infection of *Mycoplasma pneumoniae* was detected. After starting antibiotic therapy with azithromycin and doxycycline the progress could be halted and the palsies were regredient. The following MRI scans confirmed a decline in findings.

**Conclusions:**

To the authors' knowledge, this case report is the first description of SBO as an extrapulmonary *M. pneumoniae* infection. It shows the diagnostic and therapeutic complexity of a multifaceted clinical picture in which immunological, microbial and ENT-surgical diagnostic and therapeutic concepts must be regularly coordinated. Against the background of the high proportion of missing pathogens up to 30%, interdisciplinary cooperation within the framework of the ABS concept is emphasized. Structured and interdisciplinary diagnostics by a skull base center specializing in this field was ultimately decisive for treatment in this case.

## Summary

Skull base osteomyelitis (SBO) is a severe disease not just because of its rapid progression and its high mortality: diagnosis and treatment are often protracted and in more than 30% of cases no causative pathogen can be detected. SBO is usually preceded by immunodeficiency, which is why opportunistic infections caused by atypical pathogens must also be taken into consideration. The described case of an extrapulmonary *Mycoplasma pneumoniae* infection should therefore illustrate the need for interdisciplinary approaches consisting of surgical debridement, histopathological confirmation, structured microbiological diagnostics and other aspects.

## The case report

### Medical history

The 58-year-old female patient first presented in 2014 for co-evaluation with bilateral skull base osteomyelitis (SBO) of the temporal bone, which was progressive after several conservative and surgical treatment attempts. The symptoms of bilateral hearing loss (HL), putrid otorrhea and otalgia were preceded by several pneumonias requiring antibiotics. In addition, secondary immunodeficiency (SID) in the form of hypogammaglobulinemia (SHG) was diagnosed 2011 due to relapsed B-cell lymphocytic leukemia (B-CLL) and chemo-immunotherapy (with rituximab and bendamustine). Regular substitution with IVIG (Hizentra®/Cuvitru®) resulted in normal IgG and IgM titers; however, an almost complete IgA deficiency (< 5 mg/l) persisted. Despite previous therapy escalation up to bilateral petrosectomy, the existing HL continued to be progressive.

### Diagnostics

Due to the rapid progression and the still unclear etiology with a complex immunological constitution, interdisciplinary exchange to exclude various differential diagnoses took place at an early stage. In the absence of pathogen detection, a calculated antibiotic therapy with Ampicillin/Sulbactam (3 × 3 g daily) and adjuvant hyperbaric oxygen therapy (HBOT) were initiated, which had to be terminated after 7 of 20 sessions due to a reduced general condition. After stopping HBOT, the clinical and radiological findings were temporarily consistent, and the patient therefore declined further surgical revision.

In 2019, the patient presented again with bilateral progressive HL and once more putrid otorrhea. Due to radiologically increasing osseous destruction and a severe to profound hearing loss on the right ear, a revision subtotal petrosectomy with ear canals obliteration was performed on the right for rehabilitation, further diagnostics and in preparation for a two-staged cochlear implantation. Despite preoperative and preanalytical interdisciplinary case discussion, pathogen detection failed again. A local B-CLL recurrence, other malignancies, tuberculous osteomyelitis and an invasive mycosis were histopathologically ruled out in the wide range of differential diagnoses, serological tests for Treponema pallidum and brucellosis had negative results as well.

A few months later, the patient reported vertigo and an increasing contralateral hearing loss: New CT- and MRI scans confirmed osteolysis’ progression in the left temporal bone, therefore a revision of left side’s subtotal petrosectomy including the insertion of a placeholder electrode into the cochlear was performed. Several newly collected samples showed neither bacterial growth in aerobic-anaerobic culture nor mycobacterial growth in special cultures. Eubacterial and fungal Pan-PCR tests showed negative results as well, targeting a 500 bp eubacterial region of the 16S rDNA (position 26 to 527 with respect to E. coli 16S rRNA gene) and the approx. 700 bp fungal ITS region between the 18S rRNA and 28S rRNA genes [27].

Although direct pathogen detection failed once again, for the first-time epithelioid cell granulomatous formations were found in histology as a hint of a possible infectious genesis.

Despite initially good results in hearing rehabilitation with the CI on the right side, speech intelligibility with CI declined 6 months after the last surgical procedure. Radiologically, progression was confirmed on both sides once again with an especially distinct spreading of the osteomyelitis in the left temporal bone. To secure the patient’s residual hearing with CI on the right side and aiming another microbiological/pathological evidence the subtotal petrosectomy on the left side was revised a second time. (Counting surgery performed in other hospitals, it was the third revision of subtotal petrosectomy on the left side). Grasping every straw, HBOT was performed again after surgery to slow down the progression. After a steady state for another 6 weeks, a palsy of the lower group of cranial nerves (IX, X, XI nerves) with a deviation of the tongue to the left, unilateral palsy of the soft palate and vocal cord paralysis on the left appeared. Radiologically, the osteomyelitis was confirmed to have spread to the left *jugular fossa*.

As the etiology remained unclear and rapid progression seemed to continue, another interdisciplinary conference recommended the systematic exclusion of other differential diagnoses and to try another navigation-guided sampling with a focus on the diagnosis of atypical pathogens such as *Toxoplasma gondii* and *Mycoplasma pneumoniae*, which cannot be detected by standard diagnostics.

Therefore, a TaqMan-based Realtime-PCR approach was carried out targeting the B1-gene of *T. gondii* and the P1-gene of *Mycoplasma pneumoniae*. For genomic DNA extraction, a semiautomatic method was conducted using the blood and tissue kit in combination with the EZ1 Advanced machine of Qiagen (Hilden, Germany). Having a sensitivity of at least 1 to 5 genomes per reaction, the tests showed positive results for *M. pneumoniae* in four out of five specimens tested. The exact date, specimen and PCR results are listed in Table 1.

**Table 1:** Characteristics of specimen, the diagnostics method and the results

Date	Specimen	Culture	Mycobacteria culture	Pan-PCR (fungi)	Pan-PCR (bacteria)	*T. gondii*-PCR	*M. pneumoniae*-PCR
10.11.2020	Biopsy middle ear	Negative	–	–	–	–	–
	Biopsy mastoid bone	Negative	–	–	–	–	–
07.12.2021	Biopsy posterior tympanotomy	Negative	–	–	–	–	–
	Biopsy mastoid bone	Negative	–	–	-	–	–
	Biopsy tympanotomy	Negative	–	–	–	–	–
	Biopsy mastoid bone	Negative*	–	–	–	–	–
	Swab mastoid bone	Negative	–	–	–	–	–
	Swab mastoid bone left	Negative	–	–	–	–	–
	Swab mastoid bone apex	Negative	–	–	–	-	–
26.01.2022	Mastoid bone	Negative*	Negative	Negative	Negative	–	–
	Swab ear left near "electrode"	Negative	–	Negative	Negative	–	–
	Biopsy medial skull base	Negative*	Negative	Negative	Negative	–	–
	Biopsy bone medial temporomandibular joint	Negative	–	Negative	Negative	–	–
	Swab middle ear direction tube*	Negative	–	Negative	Negative	–	–
17.06.2022	Biopsy left sigmoid sinus	Negative*	Negative	Negative	–	Negative	**Positive (+/**−**)**
	Biopsy mastoid cavity left	Negative	Negative	Negative	-	Negative	**Positive (+)**
	Biopsy middle bone cranial fossa left	Negative	Negative	Negative	Negative	Negative	Negative
	Biopsy left temporomandibular joint direction	Negative	–	Negative	–	Negative	**Positive (++)**
	Biopsy middle ear left	Negative	–	Negative	–	Negative	**Positive (+++)**

–: not carried out; *after > 14 days of culturing detection of *C. acnes *(contamination not excludable); semiquantative crossing-threshold (CT-) values in the brackets

### Therapy

**Fig. 1:**
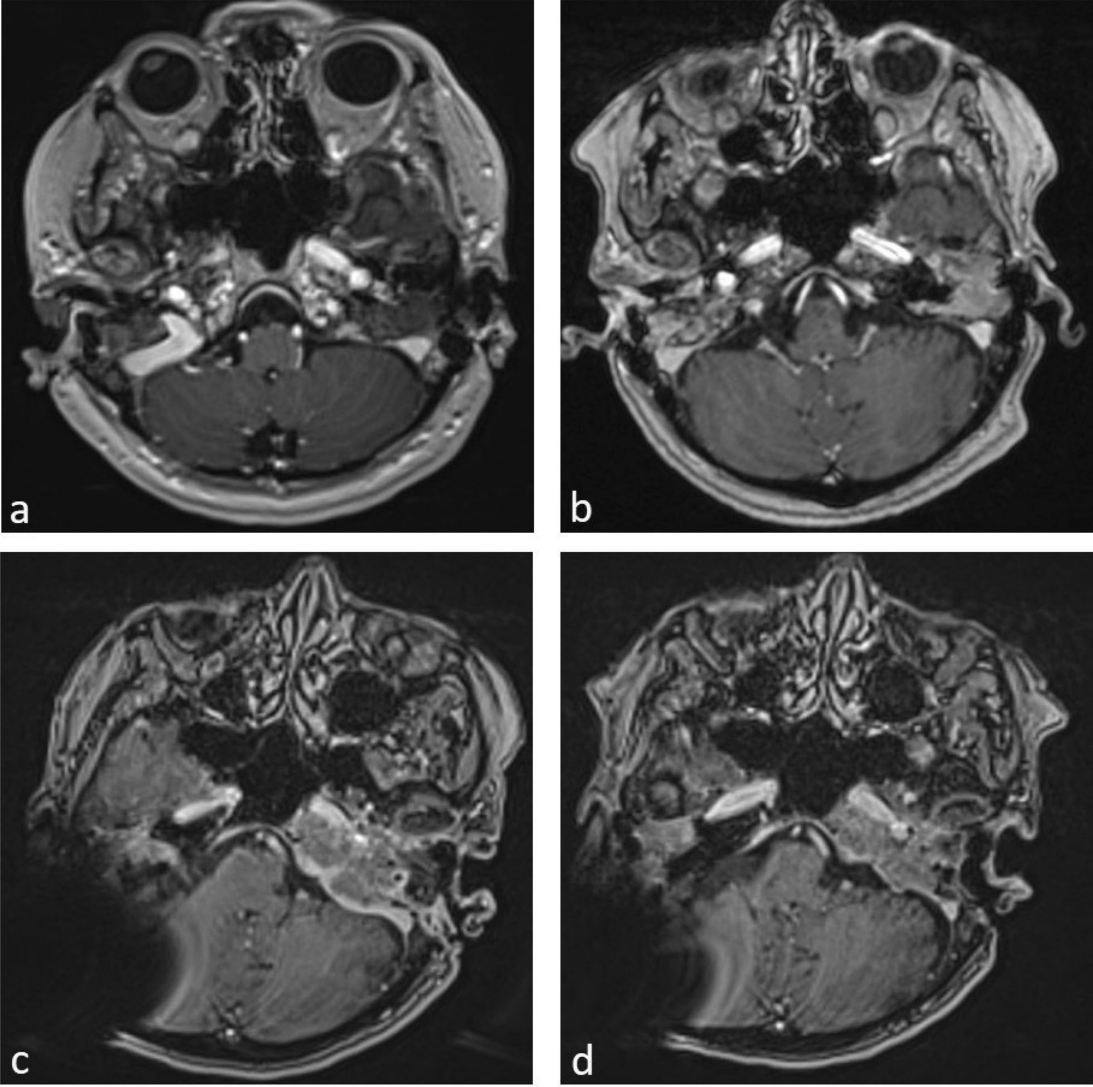
MRI imaging of the SBO’s progression at different points in time (MM/YYYY): **a** 04/2015: Initial findings. **b** 08/2019: Progressive bilateral destruction causing sensorineural hearing impairment. **c** 05/2022: Progress enclosing the left IAC, occlusion of the sigmoid sinus and spreading to the foramen magnum. *(The destruction of the right petrous bone can’t be distinguished due to the MRI signal loss after CI surgery.).* **d** 02/2024: Constancy of findings 18 months after starting antibiotic therapy

A calculated long-term antibiotic therapy with azithromycin and doxycycline was started. This gradually led to a radiological regression with sclerosis of the osteomyelitis foci and, with supportive speech therapy, to regaining mobility of the left vocal cord and palatal arch. Under close monitoring, azithromycin therapy was discontinued after 6 months and doxycycline therapy after 9 months. 12 months after diagnosis was made, the findings were morphologically consistent and clinically and audiometrically there was a clear improvement in symptoms. The patient continues to undergo close clinical, audiological and (neuro-) radiological follow-up; so far, all findings show a regression to this day. Figure 1 shows the SBO’s radiological progression at various times.

## Discussion

### Skull base osteomyelitis of the petrous bone

Skull base osteomyelitis of the temporal bone (SBO) is a serious disease due to its topographical localization and the associated high morbidity as well as the often protracted and sometimes frustrating diagnosis and treatment. Depending on the author, high mortality rates between 46% [24] and 70% [30] are described, although to the low prevalence and inconsistent terminology, etiologically different diagnoses are sometimes grouped together.

This results in a mix of different terms such as skull base osteomyelitis, petrous bone osteomyelitis, otitis externa maligna or necrotizing osteomyelitis, which are largely used synonymously depending on the author.

In the context of this case report, the terminology of SBO is used; on the one hand, to emphasize the anatomical brisance in the context of the described course of the disease, on the other hand because classical pathogenesis is often pathogen- and course-related and therefore does not seem to be applicable here.

Common to other case series is above all the clinical symptomatology with otalgia, otorrhea and increasing hearing loss as well as the protracted diagnosis, in this case of more than 8 years. Classically, the diagnosis is made in combination of clinical and radiological findings; histologically, neoplasms (e.g. lymphomas, myelomas, metastases) or immunological-rheumatological differential diagnoses (fibrous dysplasia, an inflammatory pseudotumor, an IgG-4-associated disease, Paget's disease, and various granulomatoses) should be excluded. Successful treatment requires mandatory pathogen detection [22], as a calculated, pathogen-adapted prolonged antibiotic treatment (of at least 12–48 weeks) must be carried out in addition to surgical debridement.

In recent years, the concept of antibiotic stewardship has been developed for the rational planning, implementation and re-evaluation of microbiological diagnostics and antibiotic therapy, which was also essential in diagnosing this case’s atypical infection [13]. Thanks to close interdisciplinary collaboration, it was possible to systematically narrow down the possible bacterial spectrum at the preanalytical stage by including histological findings and the previous unsuccessful antibiotic treatment attempts.

Due to previous surgery and the resulting structural defects, it became increasingly difficult to obtain new pathological and microbiological samples: In purely practical terms, it was often not possible to distinguish intraoperatively between bone osteolysis and previously introduced fatty tissue for obliteration.

We tried to minimize the risk of false-negative pathogen detection by collecting samples as standardized and evidence-based as possible [22, 23]. These minimum microbiological standards include a pre-analytical interdisciplinary consulting, a pausing of the antibiotic therapy preoperatively (in this case of 2 weeks at best [14]), the minimum number of samples required, a minimum sample size of 0.5 mm – 2 mm tissue, the avoidance of swab samples and an evaluation of the most promising transport media and conditions.

### Typical pathogen spectrum of petrous osteomyelitis

In current literature, *Pseudomonas aeruginosa*, *Staphylococcus (Staph.) aureus*, *Staph. epidermidis*, *Escherichia coli*, *Salmonella species (spp.)*, *Proteus mirabilis*, mycobacteria other than tuberculosis (MOTT), *Streptococcus pneumoniae*, *Treponema pallidum* and *Klebsiella* spp. are described as the most common bacteria causing skull base osteomyelitis. In the context of secondary immunodeficiency (SID) in particular, opportunistic pathogens such as *Mycobacterium tuberculosis*, *Brucella*, *Nocardia* or invasive mycoses have to be considered as well [2, 4, 18, 24]. In more than 30% of cases, the pathogen cannot be detected [4].

### Hypogammaglobulinemia after B-CLL

Secondary hypogammaglobulinemia (SHG) as a consequence of B-CLL has been described by various authors: According to them, the occurrence correlates with the length of disease duration, the age of the patients and various comorbidities [9, 21]. Up to 50% of deaths are caused by infections [9, 16, 20], with a particularly high percentage due to secondary immunodeficiencies. In this case, it is not clear whether the SHG is due to the B-CLL itself or to treatment with a B-cell-targeted immunotherapy (BCTT, in this case rituximab) [6, 7, 17] or the cytotoxic alkylants [28] (in this case bendamustine).

### *M. pneumoniae’s* profile

Mycoplasmas as genera of the Mollicutes are some of the smallest known prokaryotes with a size of 0.1 to 0.2 µm and a genome of only 816 kb. Due to the evolutionary loss of basic cellular mechanisms, such as cell wall synthesis, their adaptation to the human host is unique and in parts comparable to that of viruses: This explains the failure of various diagnostic (e.g. Gram or Ziehl–Neelsen stains) or therapeutic approaches (e.g. β-lactam antibiotics, glycopeptides, fosfomycin) targeting bacterial cell wall synthesis. *M. pneumoniae* is a major cause of community-acquired pneumonia (CAP) and various other extrapulmonary diseases (*M. pneumoniae* extrapulmonary diseases, MpEPDs). Similar to this case report, many systemic infections or MpEPDs in immunocompromised patients (e.g. with SID) are preceded by a previous respiratory infection [31].

### Difficulties in detecting infections with *M. pneumoniae*

Mycoplasmas cannot be visualized by light microscopy due to the lack of a cell wall, their small size (keyword: *Gram stain*) and their partially intracellular infestation [31]. Although microbiological culture is still the microbiological method of choice it cannot be recommended for the daily routine diagnosis of infections with *M. pneumoniae*:

As mycoplasma ‘s growth is inhibited by sodium polyanetholesulfonate, a major component of most commercial culture media, and specific culture media have not made it to routine diagnostics due to effort, other direct testing techniques have been established [31]. Serological tests, such as the monitoring of specific IgM, IgG or IgA titers, show low sensitivity and specificity [10]: Dumke et al. describes various immune evasion mechanisms that cause either a reduced antibody response or survival despite high antibody titers [10]. On the other hand, serological tests cannot be interpreted in the present case due to the secondary immunodeficiency and the immunoglobulin substitution. Although normal IgG and IgM titers were regularly found in the patient's immunological control serology, an almost complete absence of IgA could explain recurrent infections. Both, the particular immune evasion mechanisms and the present SID must be discussed as possible pathomechanisms in the absence of evidence [3].

This explains why nucleic acid detection [25] should be mandatory in patients with substituted hypogammaglobulinemia [15], in this case, detection was performed by 16S rDNA analysis against the P1-gene [8]. The false-negative findings of previous Pan-PCR examinations can be explained either by less sensitivity compared to the species-specific *M. pneumoniae*-PCR due to the longer length of the PCR-product, or by the complex surgical extraction of samples in the site previously obliterated with abdominal fat. The sensitivity of the P1-gene-based *M. pneumoniae* TaqMan PCR was calculated in a multi-center study by Dumke et al. [12] (in which the authors of this report took part) as one to five genomes per reaction.

### Hyperbaric oxygen therapy as an individualized treatment approach

Hyperbaric oxygen therapy (HBOT) is repeatedly discussed in case series and retrospective studies as an effective adjuvant to the antibiotic treatment of osteomyelitis [5, 18, 19, 26]. On the one hand, the increased oxygen partial pressure has positive effects by inducing neoangiogenesis and thus synergistically improving the tissue’s accessibility of antibiotic therapy, while on the other hand direct bactericidal effects are achieved, e.g. by increasing leukocyte activity [26]. In addition, both, an increase in oxygen-dependent osteoclastic resorption of necrotic bone and a stimulation of fibroblasts for collagen synthesis have been described [26].

Interestingly, *M. pneumoniae* does not secrete its own toxins, so that local destruction can be attributed to the host's own inflammatory reaction and to reactive oxygen radicals whose bacteriostatic effect is normally targeted by HBOT [29]. Knowing these therapeutic mechanisms, the limited efficacy in this case of infection with *M. pneumoniae* can be explained by its intracellular survival.

### Calculated antibiotic therapy

In treatment of *Mycoplasma* infections macrolide and tertracycline antibiotics are drugs of first choice, betalactame antibiotics fail due to the missing cell wall synthesis. Testing the bacteria’s resistance was not possible because of the missing cultural evidence. In Germany macrolide resistances are still rare (approx. 3% in pulmonary infections [11]), nevertheless taking the prolonged medical history, the vulnerable anatomy and the already taken place cranial nerve palsies into account, the decision was made to start a combine azithromycin (1 × 500 mg daily) and doxycycline (2 × 100 mg daily) consulting the ABS.

After 6 months of combination therapy, the patient was initially switched to doxycycline monotherapy due to increasing gastrointestinal ADRs. As part of the close clinical follow-up, the cranial nerve palsies continued to decline. Radiologically, the follow-up MRIs showed increasing sclerosis with decreasing CM uptake as a sign of osteomyelitis regression [1] (as shown in Fig. 1).

## Conclusion

To the authors' knowledge, this case report is the first description of SBO as an *extrapulmonary M. pneumoniae infection*. It shows the diagnostic and therapeutic complexity of a multifaceted clinical picture in which immunological, microbial and ENT-surgical diagnostic and therapeutic concepts must be regularly coordinated. Against the background of the high proportion of missing pathogens [3] up to 30%, interdisciplinary cooperation within the framework of the ABS concept is emphasized. Structured and interdisciplinary diagnostics by a skull base center specializing in this field was ultimately decisive for treatment in this case.

## Data Availability

No datasets were generated or analysed during the current study.
